# Serological Evidence of Asymptomatic Infections during *Escherichia coli* O104:H4 Outbreak in Germany in 2011

**DOI:** 10.1371/journal.pone.0073052

**Published:** 2013-09-09

**Authors:** Yanina Balabanova, Stefanie Klar, Yvonne Deleré, Hendrik Wilking, Mirko S. Faber, Sofie Gillesberg Lassen, Andreas Gilsdorf, Susann Dupke, Martin Nitschke, Friedhelm Sayk, Roland Grunow, Gérard Krause

**Affiliations:** 1 Robert Koch Institute, Berlin, Germany; 2 European Programme for Intervention Epidemiology Training, Stockholm, Sweden; 3 University Hospital Lübeck, Lübeck, Germany; 4 Helmholtz Centre for Infection Research, Braunschweig, Germany; Institut National de la Recherche Agronomique, France

## Abstract

**Introduction:**

The largest known outbreak caused by a rare hybrid strain of *Shiga toxin-producing E.coli (STEC) and enteroaggregative E. coli (EAEC) (E.coli O104:H4)* of serotype O104:H4 occurred in Germany in 2011. Fenugreek sprouts acted as a transmission vehicle and were widely consumed in the outbreak area at the time of the epidemic. In total 3,842 people developed a clinical illness caused by this strain; however the rates of asymptomatic infections remain unclear. We aimed to develop a serological assay for detection of *E.coli* O104 LPS specific antibodies and to establish the post-outbreak levels of seropositivity among people with documented exposure to contaminated sprouts.

**Results and Discussion:**

Developed serological assays (ELISA with 84% sensitivity, 63% specificity and Western Blot with 100% sensitivity, 82.5% specificity) identified 33% (16/49) level of asymptomatic infection. Relatively small sample size and a significant time- lapse between the onset of symptoms and serum samples collection (appr. 8 weeks) might explain the assay variability. No association was found between clinical or demographic characteristics and assay positivity. Larger studies are needed to understand the complexity of human immune response and factors influencing development of clinical symptoms. Development of intra-outbreak research plans will substantially aid the conduct of more thorough scientific investigation during an outbreak period.

## Introduction

From May to July 2011 the largest outbreak of severe illness characterized by haemolytic-uraemic syndrome (HUS) and bloody diarrhea affecting 3,842 people including 53 deaths occurred in Germany, primarily in its northwestern part. The outbreak was caused by a rare hybrid strain of *Shiga toxin-producing E.coli (STEC) and enteroaggregative E. coli (EAEC) of serotype O104:H4 (E.coli O104:H4)* [1]–[3] with an extended spectrum of β-lactamase resistance. The outbreak was associated with a number of non-typical features such as older age of patients who were mainly women, a higher proportion of patients developing HUS and a high incidence of neurological symptoms. Fenugreek sprouts were identified as the most likely transmission vehicle [4]–[6]. It can be assumed that the sprouts were widely consumed in the outbreak area at the time of the epidemic; however, only a number of people developed clinical illness. It is unclear whether some of the exposed individuals who remained healthy were also infected.

An infection with toxin producing *E.coli* bacteria triggers the production of serum antibodies directed against the lipopolysaccharides (LPS) of the pathogen [7]. Therefore measurement of antibodies against LPS by serological assays such as ELISA and Western Blot (WB) has been widely used for diagnosis of a number of illnesses caused by gram-negative organisms including *E. coli* O157:H7 [7]–[9]. However, at the time of the outbreak an assay able to give evidence of the antibody status after an infection with the outbreak strain was not available.

We aimed to develop and validate a serological assay for detection of *E.coli* O104 LPS-specific antibodies and to establish the post-outbreak levels of seropositivity among a group of people with documented exposure to the contaminated sprouts.

## Methods

### Ethics Statement

The study received an approval from the Charité University Clinics Ethics Committee, Berlin. All study participants provided a written informed consent to participate in the study.

### Assay development

Samples obtained from patients with culture-confirmed *E.coli* O104:H4 infection (positive control group) and individuals assumed not to be infected with this *E.coli* strain (negative control group) were used for the assay validation. The culture-confirmed patients were followed up by the Lübeck University clinic (n = 31; 21 were female and 28 presented with HUS). The median time from the symptom onset to the blood sampling was 8 weeks (range: 5 to 9 weeks). A group of negative controls (n = 473) included residual samples obtained from blood donors residing in a non-outbreak area (n = 444) and archived samples (n = 29) from patients with culture-confirmed *E.coli* infections of serotypes other than O104:H4 (O3, O26, O91, O103, O111, O145 and O157). The latter specimens were collected within the framework of large population-based surveys and were used for testing a possible cross-reactivity. All samples were initially screened by the developed specific ELISA assay; the results of all samples from cases infected with non-outbreak strains and of a proportion of the donors' samples (all samples detected by ELISA as positive, weak positive and a random number of negative samples) were subsequently tested by a confirmatory WB assay able to visualize the LPS diversity among the *E.coli* serotypes using O104 LPS as an antigen [10]. ELISA was conducted according to the LPS-ELISA method as published elsewhere [11]. The outbreak strain *E.coli* O104:H4 was used for LPS preparation (Micromun, Greifswald, Germany) and utilized at a concentration of 1 µg/ml for coating.

For the WB assay specific LPS profiles were prepared by SDS-PAGE [12]. Purified O104 LPS (80 µg/gel) was loaded onto preparative gels comprising 4% (w/v) acrylamide stacking gel and 12% (w/v) acrylamide separation gel. Electrophoresis was conducted using the BioRad Mini Protean 3 (BioRad, Munich, Germany) system with constant current of 40mA/gel. The separated LPS was transferred onto a PVDF-membrane (Immobilion, Millipore, Schwalbach, Germany) with 1 mA/cm^2^ for 1h. Subsequently the membrane was blocked with 10% milk powder (in TBS-T) and cut into stripes, which were then incubated with 1:500 or 1:1000 dilutions (in blocking buffer) of the corresponding sera. Antigen-antibody complexes were detected using a goat anti-human polyvalent immunoglobulin, conjugated with horse-radish peroxidase (Dianova, Hamburg, Germany), 0.2 µg/ml in blocking buffer, and TMB (Seramun, Heidesee, Germany) as substrate. Antibody binding was assessed by the intensity of the immunoblot reaction and the LPS profile. Only serum samples that generated the specific O104 LPS profiles were considered as positive for a recent O104:H4 infection linked to the outbreak. In order to establish the dilution of sera that provide the best specificity of the Western Blot assay several negative control samples previously tested in ELISA as negative or positive were titrated; the best discriminatory dilution was found at 1:1000 when all positive control sera also showed a positive result.

### Study population

To evaluate the levels of seropositivity we invited individuals with documented exposure to the contaminated sprouts to take part in the study. These were the members of the three largest visitor groups who had meals containing contaminated sprouts at a particular restaurant (“restaurant cohorts”). Following the meal a large number of them developed a gastroenteritic illness while the rest stayed clinically healthy. Exposure to the sprouts was documented for each individual through a careful examination of the menus, interviews with the group members and a restaurant chef as well as with an aid of available photos of the dishes made by the restaurant visitors. Nearly half of the dishes served at the restaurant during the period of the outbreak contained sprouts that were purchased from one distributor [5]. This detailed epidemiological investigation provided the key evidence supporting the fenugreek sprouts being a transmission vehicle and aided implementation of adequate control measures.

A serum sample was taken from each participant for laboratory examination after 6-9 months after the onset of the symptoms.

## Results

### Assay development and validation

Of 31 samples from the positive control group, 28 were determined as positive and 3 as borderline by the ELISA resulting in a sensitivity of 100% (when the borderline results are interpreted as being positive). The ELISA identified 175 samples from the negative control group as false positive thus demonstrating inadequate specificity of 63%.

The WB assay confirmed all 31 positive control samples as positive (sensitivity 100%); however 17.6% (19/108) of the blood donors' specimens and 17.2% (5/29) of the specimens from patients infected by non-outbreak strains were also falsely tested as positive corresponding to an overall moderate specificity of 82.5% (Table 1).

**Table 1:** Performance characteristics of Western Blot in detecting *E.coli* O104:H4 antibodies.

Test characteristics	n/N (%)	95%CI
Sensitivity	31/31 (100)	88.8–100
Specificity overall	113/137 (82.5)	75.1–88.4
Specificity: blood donors only	89/108 (82.4)	–
Specificity: patients infected with non-O104:H4 STEC serotypes only	24/29 (82.8)	–
LR+	5.7	4.0–8.2
LR−	0	–

LR+ – likelihood ratio of a positive test; LR- -likelihood ratio of a negative test.

*E. coli- Escherichia coli.*

It is known that the O104 LPS of *E.coli* is structurally closely related to the capsular K9 polysaccharide of *E.coli* [13], which is only expressed with O groups 8 and 9 serotoypes of *E.coli*. To check for this possible cross-reactivity several O104:H4 positive serum samples were first tested against the following strains: O9:K9:H12; O8:K9:H19; O9:K9:H19. All tested sera reacted with the O9 strains (data not shown).

Culture-confirmed patients infected with *E.coli* of other than O104:H4 serotypes were then tested in a two-step procedure. In the first batch of samples of non-O157 serotypes (n = 9) the results for all samples were negative. In the second batch of O157 serotypes (n = 20) only four reacted with the O104 LPS in the WB assay and in one sample the reaction was borderline positive. In summary no appreciable cross-reactions were observed with non-O157 serotypes sera, whereas 25% results of the O157-serotype group were false positive.

### The rates of infection

Two-third of the invited persons (66/95; 69%) agreed to participate in the study (67% female, median age 58 years, interquartile age 47–70 years). All but two participants reported sprout consumption. Comparable proportion of those who took part in the study (17/66; 26%) and those who did not due to difficulties related to the travel to the blood collection point (6/29; 21%) met the outbreak case definition [5]. Of the 14 culture confirmed cases 8 (57%) were tested positive (Table 2): two persons were severely ill with HUS and the remaining 6 had bloody diarrhea without HUS. Two culture-negative patients were also serologically positive. Of the 6 culture-confirmed patients who were serologically negative, two had HUS and four had bloody diarrhea only.

**Table 2:** Positivity rates identified by the Western Blot (WB) assay among restaurant cohorts' participants (n = 66).

Category of participants		WB+	WB−	Positivity rate%
Cases*		10	7	59
	Culture confirmed case	8	6	57
	No culture confirmation	2	1	67
Non-case		16	33	33
	Sprout consumption	16	31	34
	No sprout consumption	0	2	0
All cohorts' members		26	40	39

* All patients meeting the outbreak case definition including culture-negative cases (a case was defined as a patient with clinically diagnosed hemolytic-uremic syndrome, bloody diarrhea and/or culture confirmation).

Of note, blood samples were taken from the restaurant cohorts' members significantly later than from the patients used as positive controls for the assay validation. The delay was related to the need to finish outbreak investigations, to liaise with treating physicians and potential study participants and to the time necessary for obtaining an ethic approval for the study.

One third (33%) of the cohort members who were not clinically ill were tested positive. Only two cohort members did not consume sprouts and none of them was serologically positive. None of the known clinical or demographic factors (gender, age, clinical severity of illness) were associated with positivity on a univariate analysis; however the numbers were small to draw significant conclusions. The rate of seropositivity detected by the WB among the asymptomatic sprout-consumers (34.0%; 16/47) was significantly higher (p = 0.018) than among the negative control group (17.5%; 24/137).

## Discussion and Conclusions

Within the framework of the study two consecutive serological assays able to identify an infection with *E.coli* O104:H4 that had caused the largest known outbreak in Germany in 2011 were successfully developed. The development and validation procedures were based on the known LPS antigenic status of gram negative pathogens and the specific LPS structures of the outbreak strain.

The validation data clearly showed the applicability of the ELISA as a screening tool for testing a large number of samples; a further testing by the WB assay is necessary to confirm the diagnosis in ELISA-positive individuals although the results need to be interpreted with caution and in conjunction with the epidemiological data on exposure.

Fewer cohorts' members who had been ill were identified as serologicaly positive than the culture-confirmed patients from the positive control group. Observed variability in the assay ability to detect immunological responses might depend on the kinetics of the antibodies formation, their decreasing stability and regression against the O104 antigen due to the waning immunity phenomenon since a longer period of time elapsed between the onset of symptoms and the sampling in the latter group (6–9 months compared to the 6 weeks in the positive control group). Previously reported data show that *E. coli* serotype O157 fails to induce a lasting humoral immune response to the LPS O-antigen in children with HUS leading to decrease in the ELISA signal 5–6 weeks after onset on diarrhea and a level of IgM and IgG antibody below the respective cutoff levels after 11 weeks [10]. Indeed the antibody titer of the positive patients in the study group was lower than in the positive control group.

The study data suggest that a significant proportion of people who remained clinically healthy were infected with *E.coli* O104:H4 after being exposed to contaminated sprouts during the outbreak in Germany in 2011. Estimated likelihood ratio of a positive test provides moderate evidence [14] that the positive assay results are indeed associated with an infection by *E. coli* O104:H4. However, even accounting for imperfect specificity and a 17.5% chance of a false-positive result, at least 28% positivity rate among exposed but not ill cohort members can be truly expected. Possible relation between the exposure level and the intensity of clinical symptoms might explain why a number of people remained healthy despite consumption of the contaminated sprouts. Although the WB assay has an excellent sensitivity we cannot rule out that not all infected but clinically asymptomatic individuals were detected since some of them might have had a low antibody response below the detection limit.

It is known that people can be symptomless carriers of a pathogen. The detection of serum antibodies to the LPS of O157 in apparently healthy individuals with occupational contact with cattle suggested that exposure to low levels of the pathogen may stimulate an immune response to the organism [7].

High positivity rate among negative controls could possibly be explained by the prevalence of unrecognized infections caused by other low pathogenic *E.coli* strains, as it is known, for example that the O104 LPS and the capsular K9 polysaccharide found in *E.coli*-serotypes O8 and O9 share structural identities [13]. Both strains are linked to urinary tract infections and septicemia [15]. Indeed we have identified significant interactions of O104 samples with the O9 serotype of *E.coli*; however the nature of the cross-reactivity should be investigated further. In addition, developed assays detect all infections caused by the O104 serogroup like e.g. O104:H21 which is known as the pathogen responsible for a small outbreak in Helena, Montana in 1994 [16]. Little is known about the natural appearance and infection incidence of the *E.coli* O104 serogroup in Germany but it can be assumed that this serogroup has low distribution.

For the O157 LPS of *E.coli* it has been shown that it shares epitopes with the LPS from other bacterial pathogens, as e.g. *Yersinia enterocolitica* or *Brucella spp.* [7]. Although current surveillance data demonstrating low rates of infection with these pathogens [17] making this explanation unlikely, it may be true that O104:H4 cross-reacts with other gram- negative pathogens thus resulting in imperfect specificity of the assay. Cross-reaction of the tested O157 samples with the O104 LPS in a quarter of the cases indicates that some epitopes remain similar between the O157 and O104 O side chains.

The study was limited to the convenience sample of culture-positive patients used for the validation purposes and to a relatively small number of ill and healthy but exposed persons from the restaurant cohorts thus permitting spectrum bias. We did not find evidence to show that any of the clinical or demographic characteristics were associated with being positive on the assay.

Accounting for imperfect test parameters, the test can be probably recommended to be used in people with suspected severe *E.coli* infection and HUS, especially in the absence of positive culture confirmation but it is of a less value in identifying contacts of infected people who are not severely ill.

Larger studies are needed to understand the complexity of human immune response, risk factors for an initial infection as well as factors influencing development of a clinical illness and the severity of the symptoms. However small, this study might guide the further research geared towards a better understanding of susceptibility to the EHEC infections and the development of rapid and accurate diagnostic tools.

One important conclusion relates to logistical difficulties in conducting post-outbreak research. Hence development of “intra-outbreak” research plans describing standard methodological approaches to samples collection, data protection, ethical issues and mechanisms for rapid pre-approval of studies, setting-up common databases and information exchange mechanisms is prompting. This plan could enable better quality research resulting into evidence-based findings. In the time of a large outbreak when all clinical, laboratory and epidemiological resources are mobilized a clear algorithm of research-related actions is needed.
